# Impact of emergency physician performed ultrasound in the evaluation of adult patients with acute abdominal pain: a prospective randomized bicentric trial

**DOI:** 10.1186/s13049-024-01182-5

**Published:** 2024-02-26

**Authors:** François Brau, Mathilde Papin, Eric Batard, Emeric Abet, Eric Frampas, Aurélie Le Thuaut, Emmanuel Montassier, Quentin Le Bastard, Philippe Le Conte

**Affiliations:** 1 Service des urgences, Centre Hospitalier Départemental, La Roche-Yon, France; 2 Service Des Urgences, Centre Hospitalier Universitaire, 44035 Nantes Cedex 01, France; 3 Faculté de Médecine, Nantes Université, Nantes, France; 4 Service de Chirurgie Digestive, Centre Hospitalier Départemental, La Roche-Yon, France; 5 Service de Radiologie, Centre Hospitalier Universitaire, Nantes, France; 6 Plateforme de Méthodologie et Biostatistique, electriqueDirection de la Recherche Et de L’Innovation, Centre Hospitalier Universitaire, Nantes, France

**Keywords:** Abdominal pain, Diagnostic, Point-of-care ultrasound, Emergency medicine

## Abstract

**Background:**

Abdominal pain is common in patients visiting the emergency department (ED). The aim of this study was to assess the diagnostic contribution of point-of-care ultrasound (POCUS) in patients presenting to the ED with acute abdominal pain.

**Methods:**

We designed an interventional randomized, controlled, open label, parallel-group, trial in two French EDs. We included adult patients presenting to the ED with acute abdominal pain. Exclusion criteria were a documented end-of-life, an immediate need of life-support therapy and pregnant or breast-feeding women. Patients were randomized in the experimental group (i.e., workup including POCUS) or control group (usual care). The primary objective of the study was to assess the added value of POCUS on diagnostic pathway in the ED, according to the diagnostic established a posteriori by an adjudication committee. The primary endpoint was the proportion of exact preliminary diagnosis between the 2 groups. The preliminary diagnosis made after clinical examination and biological results with POCUS (intervention arm) or without POCUS (usual care) was considered exact if it was similar to the adjudication committee diagnosis.

**Results:**

Between June 2021 11th and June 2022 23th, 256 patients were randomized, but five were not included in the primary analysis, leaving 125 patients in the POCUS group and 126 patients in the usual care group (130 women and 121 men, median [Q1-Q3] age: 42 [30;57]). There was no difference for exact diagnosis between the two groups (POCUS 70/125, 56% versus control 78/126 (62%), RD 1.23 [95% CI 0.74–2.04]). There was no difference in the accuracy for the diagnosis of non-specific abdominal pain nor number of biological or radiological exams. Diagnostic delays and length of stay in the ED were also similar.

**Conclusions:**

In this trial, systematic POCUS did not improve the rate of diagnostic accuracy in unselected patients presenting to the ED with acute abdominal pain. However, as it was a safe procedure, further research should focus on patients with suspected etiologies where POCUS is particularly useful.

*Trial registration: *This trial was registered on ClinicalTrials.gov on 2022/07/20 (https://clinicaltrials.gov/study/NCT04912206?id=NCT04912206&rank=1) (NCT04912206).

## Background

Non-traumatic abdominal pain is one of the most common complaints in patients visiting the emergency department (ED) [1]. It represented 6.5% of patients admitted in a US ED in 2007 [1]. Many etiologies may be involved in adult patients presenting with acute abdominal pain: surgical (appendicitis, bowel obstruction…), medical (diverticulitis, cholelithiasis, gastritis, renal colic, urinary tract infections …) but also non-specific abdominal pains [2]. It therefore remains a daily diagnostic challenge for emergency physicians (EP). Diagnosis accuracy relies on improvements in the advanced imaging tools such as computed tomography (CT) or ultrasound (US) [1, 3]. Currently, the diagnostic workup in a patient presenting with acute abdominal pain, is based on clinical examination and if necessary, laboratory tests and, in many cases, imaging procedures.

Beside ultrasound performed by the radiology department, point-of-care ultrasound (POCUS) is increasingly used to assess patients in emergency medicine (EM) for many years [4]. It has become an integrated part of the EM curriculum [5]. US is particularly suited to assess patients with acute abdominal pain as many organs are easily explored [6]. Diagnostic performances, both sensitivity and specificity, are higher for cholelithiasis, renal colic and small bowel obstruction when compared with other illnesses such as diverticulitis. Lindelius demonstrated that a surgeon-performed US was able to increase diagnosis accuracy in patients with acute abdominal pain [7]. Furthermore, in this same study, POCUS decreased short-term complementary examinations [8] and increased patient’s satisfaction. It was demonstrated that POCUS could decrease the hospitalization rate ( − 7%), imaging prescriptions ( − 18%) and increase the direct orientation toward surgery by 18% without increasing rates of rehospitalization or death [9]. Another study showed that POCUS increased the diagnostic accuracy and planned diagnosis workup by 45% [36–54%] in a population of 128 patients admitted to the ED with abdominal pain without previous diagnostic orientation [10]. This improvement was achieved by comparing diagnosis before and after POCUS realization. POCUS can also be used as a first-line imaging procedure followed by a computed tomography if necessary. This strategy has been considered as the most accurate according to sensitivity and exposure to radiation [2]. POCUS could therefore be considered as an extension of the clinical examination and can increase the whole diagnostic performance, in particular in some illnesses as cholelithiasis or renal colic.

However, despite these few studies, the added value of POCUS performed by an emergency physician on patients with non-selected abdominal pain remains controversial. Actually, (i) it has rarely been evaluated by randomized controlled trial (RCT), (ii) US performances have only been demonstrated for some conditions such as renal colic, cholelithiasis, appendicitis or bowel obstruction and (iii), previous studies were performed by highly trained Emergency Physicians.

Here, we conducted a RCT to investigate the added value of early POCUS on the accuracy of the preliminary diagnosis made by the emergency physician before any radiologist-performed imaging study in patients presenting to the ED with acute abdominal pain [11].

## Methods

### Design

We designed an interventional randomized, controlled, open label, parallel-group, trial in two French EDs. The two recruiting centers were a university hospital and a community hospital. POCUS was added to the usual diagnosis workup in the experimental group. POCUS, performed in B mode only with a curvilinear probe, assessed the major spots and search for main anomalies. This study followed the CONSORT reporting guidelines [12].

### Intervention

Since there is no international consensus-based guideline, the exploration protocol was collectively designed after a literature review. It was focused on aorta (aneurysm, aortic dissection), gallbladder (lithiasis, cholecystitis), kidneys (hydronephrosis), bladder, small bowel loops, appendix and ovaries (Table 1). POCUS was performed using Mindray TE7 or Philips CX50 with a curvilinear probe (3.5–5 MHz) or a linear probe (7–10 MHz) for the appendix exploration. It was performed by a trained EP. Theses EP previously attend a refresher course [12], and were not necessarily the EP in charge of the patient. The investigators have previously completed a validated training program. It could be a certified one-year faculty-based training program or a short training session (two days). The study protocol was published [11].

**Table 1:** Spots and focused anomalies visualized by POCUS in the echoPAIN study

Organ	Pathological finding (illness)
Abdominal aorta	Dilation (Aneurysm), flap (aortic dissection)
Gallbladder	Cholelithiasis Murphy sign, wall thickening (cholecystitis)
Kidneys	Hydronephrosis (renal colic)
Bladder	Dilation (urine retention)
Peritoneum (pouch)	Presence of fluid
Small bowel loops	Dilated, incompressible loops with back-and-forth liquid movement (bowel obstruction)
Appendix	Non-compressible appendix with diameter > 6 mm (appendicitis)
Ovaries	Ovarian cysts or mass

### Participants

We included patients strictly over 17 years old presenting to the ED with acute abdominal pain, when an EP trained in POCUS was present. They were identified from the referral system. An informed consent was obtained before randomization. It was a convenience sample since an EP trained in POCUS was not always available. Exclusion criteria were a documented end-of-life, immediate need of life-support therapy, pregnant or breast-feeding women and patient under guardianship.

### Outcomes

The final diagnosis was established a posteriori by an adjudication committee composed of three independent experts in EM, radiology and abdominal surgery. The committee was blind regarding the group (POCUS vs control). They had access to all data from patient files including advanced imaging results excepted the preliminary diagnosis made by the emergency physician in charge of the patient and POCUS results. The preliminary diagnosis was made by the treating EP before any radiologist-performed imaging study (including ultrasound, CT-scan, MRI). The preliminary diagnosis was based on clinical examination and results of laboratory tests in both arms, associated with POCUS in the intervention arm. The treating EP were not blind of the POCUS results in the intervention arm.

The primary endpoint was the proportion of exact preliminary diagnosis. The preliminary diagnosis made by the EP was considered exact/correct when it was similar to the final diagnosis made by adjudication committee. Diagnosis were chosen in a predefined list. The diagnostic criteria were not specified since the adjudication committee was composed of experts. Non-specific abdominal pain was defined as an acute abdominal pain of under 7 days’ duration, and with no diagnosis after examination and baseline investigations. Secondary endpoints were the time between admission at the ED and diagnosis, ED length of stay, diagnostic accuracy for non-specific abdominal pain, prescription of biological and radiological exams during the ED length of stay and hospitalization rate. A post-hoc analysis on diagnostic performance was performed on a sub-group of patients with diagnosis accessible to US.

### Randomization

Patients were randomized 1:1 to POCUS or control group by a computed-based program in random block sizes and stratified by centre. Randomization list were generated using SAS software.

#### Sample size calculation

Based on previous studies [7–10], a correct diagnostic rate of 57% was expected in the control group and 74% in the experimental group. With an alpha value of 0.05 and a power level of 80%, 244 patients were required. A 5% attrition rate (patients randomized but presenting an exclusion criteria) was expected, thus 256 randomized patients were needed.

#### Statistical analysis

The primary endpoint was compared between the two groups using a mixed model taking into account the recruiting centre. The delays were compared by a mixed linear generalized model adjusted on the recruiting centres. Sensitivity, specificity, positive and negative predictive values were estimated with their 95% confidence intervals. The rates of readmission and hospitalization were compared using a logistic generalized mixed model adjusted on the centre. In the experimental group (with POCUS), duration and self-assessed difficulty of POCUS were described by means and standard deviations.

## Results

**Fig. 1:**
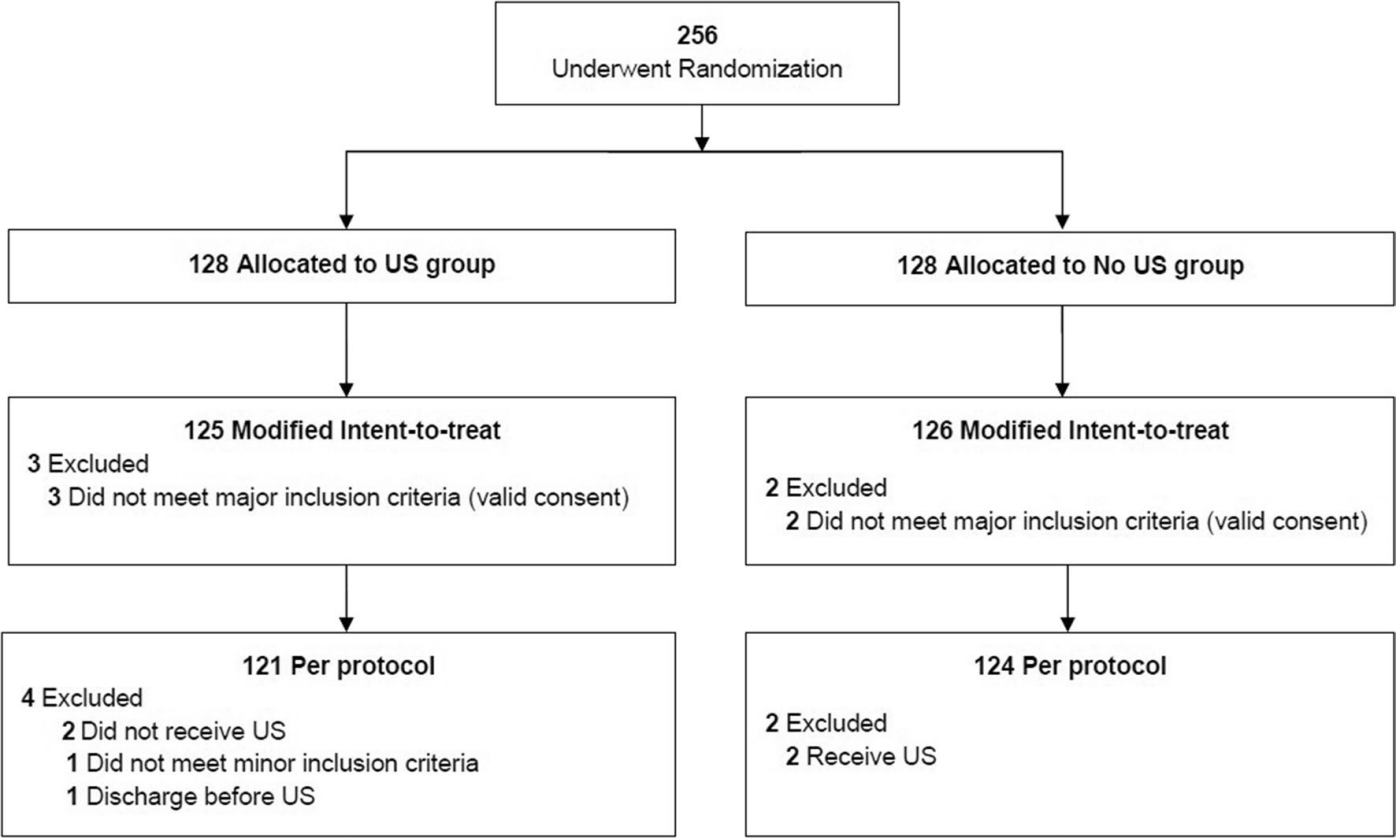
Flow-chart of 256 patients included in the study

Between June 2021 11th and June 2022 23th, 256 patients were randomized, but five were not included in the primary analysis (lack of valid consent), leaving 125 patients in the POCUS group and 126 patients in the usual care group (Fig. 1). Baseline characteristics are displayed in Table 2. Briefly, there was 130 women and 121 men, median [Q1-Q3] age: 42 [30;57]. Baseline characteristics were similar between the 2 groups. Pain visual analogic scale was 7 [5–8] in POCUS group and 7 [5–8] in the control group (median [Q1-Q3]). According to the adjudication committee, the most frequent diagnoses were: non-specific abdominal pain (69 patients, 28%), renal colic (30 patients, 12%), diverticulitis (18 patients, 7%), gastroenteritis (18 patients, 7%), cholecystitis (17 patients, 7%), appendicitis (16 patients, 6%), pyelonephritis (15 patients, 6%) and cholelithiasis (14 patients, 5%).

**Table 2:** Baseline characteristics and final diagnosis of 251 patients included in the study

		POCUS group N = 125	usual care group N = 126	Total N = 251
Age	Median [Q1;Q3]	44 [31;61]	40 [28;55]	42 [30;57]
Sex	Male	60 (48%)	61 (48%)	121 (48%)
	Female	65 (52%)	65 (52%)	130 (52%)
BMI	Mean ± SD	26 ± 5	25 ± 5	25 ± 5
Abdominal comorbities		35 (28%)	37 (29%)	72 (29%)
Cardial comorbidities		11 (9%)	13 (10%)	24 (10%)
Duration of pain (h)	Median [Q1;Q3]	12.0 [5.00;48.00]	19.0 [5.50;54.00]	14.0 [5.00;48.00]
VAS (pain)	Median [Q1;Q3]	7.0 [5.00;8.00]	7.0 [5.00;8.00]	7.0 [5.00;8.00]
Tenderness		26 (21%)	27 (21%)	53 (21%)
Rigidity		3 (2%)	5 (4%)	8 (3%)
‍Non specific abdominal pain		38 (31%)	31 (25%)	69 (28%)
‍Renal colic		15 (12%)	15 (12%)	30 (12%)
‍Diverticulitis		11 (9%)	7 (6%)	18 (7%)
‍Gastroenteritis		7 (6%)	11 (9%)	18 (7%)
‍Cholecystitis		7 (6%)	10 (8%)	17 (7%)
‍Appendicitis		4 (3%)	12 (10%)	16 (7%)
‍Pyelonephritis		99 (7dd	6 (5%)	15 (6%)
‍Cholelithiasis		9 (7%)	5 (4%)	14 (6%)

*BMI* Body mass index, *VAS* Visual assessment scale

Modified intend-to-treat analysis showed no difference for the exact diagnosis between the two groups (POCUS group: 70/125, (56% [95% CI 47–64%]) versus usual care group: 78/126 (62% [95% CI 53–70%]), RD 1.23 [95% CI 0.74–2.04]). NPV and PPV in the POCUS group were 79% [69%; 86%] and 62% [42%; 79%] respectively, and in the control group, 84% [75%; 90%] and 53.6% [33.9%; 72.5%] respectively. There was no difference in the accuracy for the diagnosis of non-specific abdominal pain between POCUS and control groups, sensitivity 47% versus 48% and specificity 87% versus 86%, respectively. There was no difference in numbers of laboratory tests, CT and US performed by the radiologists (Table 3). Time from door to diagnosis were 3.8 + 2.2 and 4.0 + 2.5 h in POCUS and control groups, respectively (*p* = 0.78). ED length of stay ED were 9.9 + 6.5 h and 10.0 + 6.3 h in POCUS and control groups, respectively (*p* = 0.57). Finally, there was no difference between the two groups for hospitalization rate (POCUS group: 38/123, (31% [95% CI 23–40%]) versus control group: 45/126 (36% [95% CI 28–44%]), absolute risk difference (RD) 0.8 [95% CI: 0.47–1.37]) nor for readmission to the ED at day-7 (POCUS group 3/113, 3% [95% CI 0.6–8%]) versus control (3/111, 3% [95% CI 0.6–8%]), RD 0.98 [95% CI 0.19–5.02]). Duration of POCUS was 8 + 4 min and the self-assessed difficulty on a Likert scale ranging from 1 (very easy) to 10 (impossible) was 2 + 2.

**Table 3:** comparison of secondary outcomes in 248 patients included in the study (3 patients with missing data)

Secondary endpoints	With POCUS n = 123	Without POCUS n = 125	Absolute risk difference [95%CI]	*p*-Value
Number of laboratory tests	4.2 ± 1.4	4.2 ± 1.4	0.01 [ − 0.31; 0.34]	0.93
Number of CT	52/123 (42.3%)	47/126 (37.3%)	4.97 [ − 7.72;17.12]	0.37
Number of US performed by a radiologist	39/123 (31.7%)	37/126 (29.3%)	2.34 [ − 9.10;13.78]	0.70
Time from door to diagnosis	3.79 ± .18	3.98 ± 2.50	− 0.19 [ − 0.80;0.43]	0.78
ED length of stay	9.92 ± 6.56	9.96 ± 6.31	− 0.04 [ − 1.65;1.56]	0.57
Hospitalization rate	38/123 (30.89%)	45/126 (35.71%)	− 4.82 [ − 16.51;6.87]	0.42
Readmission to the ED at day-7	3/113 (2.65%)	3/111 (2.70%)	− 0.05 [ − 4.28;4.18]	0.98
Duration of POCUS	8.12 ± 3.38			
Self-assessed difficulty	2.43 ± 2.46			

*CT* Computed tomography, *US* Ultrasound, ED: Emergency department, POCUS: Point-of-care Ultrasound

In the subgroup of patients with cholelithiasis, cholecystitis, renal colic or bowel obstruction, the diagnostic accuracy was 25/35 (71% [95%CI 47–64%]) in the POCUS group and 22/33 (66% [95% CI 50–80%]) in the control group (*p* = 0.74).

## Discussion

In this bicentric randomized controlled study, systematic POCUS performed by an EP did not improve the rate of exact diagnosis in adult patients presenting to ED with acute abdominal pain. This particular endpoint, rate of exact diagnosis in patients with unselected abdominal pain, has not been frequently addressed. Lindelius [7] showed a positive effect on diagnostic performance with an improvement of exact preliminary diagnosis from 57 to 65%. In a population with similar inclusion criteria, Durgun [13] found that POCUS was able to narrow the number of suspected diagnoses, to reduce the ED length of stay, but not the whole cost. When comparing population between our study and the one from Lindelius, there was no obvious differences. Distribution of final diagnosis and diagnostic accuracy in the control group, were similar. The lack of positive effect of POCUS on diagnostic accuracy could be explained by several factors: (I) insufficient POCUS skills of investigators. They were diverse even if a validated training session was required to be an investigator. However, duration and modalities of these training programs could be different” In the study from Lindelius, surgeons had a 4-week training session which is longer than the ones of some of our investigators. (ii) POCUS could be performed by an investigator who was not the EP in charge of the patient. Therefore, US findings were possibly not fully integrated with other clinical findings. In addition, we did not observe a decrease in US performed by a radiologist ordered by the emergency physician in charge of the patient in the POCUS group compared to the control group. This result contrasted with Lindelius study in which less radiological US were performed in the PoCUS group. It could be related to the lack of integration of PoCUS findings in the diagnostic process or to the lack of confidence of investigators.

Other studies investigated the diagnostic accuracy of POCUS in specific presentations such as suspected bowel obstruction [14, 15], acute flank pain [14], suspected appendicitis [15]. In all these studies, POCUS improved the diagnostic accuracy. Another approach was the diagnosis or management changes when POCUS was performed. Jang [10] showed that POCUS improved the decision making process by 45% [CI 95% 36–54%].

Our study had some limitations: (I) varied POCUS skills of investigators; (ii) Overestimation of the potential diagnostic improvement induced by PoCUS, (iii) The experts reviewed the patient’s file together which could introduce bias and (iv) US clips quality were not adjudicated since it should have require a full review process.

Despite these limitations, it was a prospective randomized control study without major deviation. Moreover, POCUS was harmless as there was no difference in safety criteria (same hospitalization and 7-day readmission rates between the two groups). Indeed, abdominal US, including POCUS, only provides useful insights in some etiologies (cholelithiasis, renal colic, appendicitis, small bowel obstruction). This could explain the modest but real POCUS effect in the study from Lindelius (7%) and its absence in the whole population of the current one. A potentially interesting strategy could be a clinical evaluation followed by a POCUS only in case of suspected diagnoses accessible to POCUS. A CT would be required in case of inconclusive POCUS or clinical situation requiring definite diagnosis.

## Conclusion

Our study did not demonstrate a positive effect of POCUS in the diagnostic process of adult patients with abdominal pain. Other diagnostic strategies including PoCUS could be tested for patients consulting for acute abdominal pain. Further research should focus on the accuracy of strategies including PoCUS only if an etiology where it is accurate, are suspected. This approach was investigated with some success [2] but still need confirmation [16].

## Data Availability

The datasets generated and/or analyzed during the current study are not publicly available due to protection of study participant privacy but are available from the corresponding author on reasonable request.

## Abbreviations

POCUS: Point-of-care ultrasound
ED: Emergency department
EP: Emergency physicians
CT: Computed tomography
US: Ultrasound
EM: Emergency medicine
ECRFs: Electronic case report files
